# Supplementary material for: Neutralizing and interfering human antibodies define the structural and mechanistic basis for antigenic diversion
Source: Nat Commun. 2022 Oct 6;13:5888. doi: 10.1038/s41467-022-33336-3 (PMC9537153; doi:10.1038/s41467-022-33336-3)
Supplement: Supplementary file 1 — Supplementary Information [file 41467_2022_33336_MOESM1_ESM.pdf]

Supplementary Information for:

Neutralizing and interfering human antibodies define the structural and mechanistic basis for antigenic diversion

Palak N. Patel[1], Thayne H. Dickey[1], Christine S. Hopp[2], Ababacar Diouf[3], Wai Kwan Tang[1], Carole A. Long[3], Kazutoyo Miura[3], Peter D. Crompton[2], Niraj H. Tolia[1]*

[1]Laboratory of Malaria Immunology and Vaccinology, National Institute of Allergy and Infectious Diseases, National Institutes of Health, Bethesda, MD, USA

[2]Malaria Infection Biology and Immunity Section, Laboratory of Immunogenetics, National Institute of Allergy and Infectious Diseases, National Institutes of Health, Rockville, MD, USA

[3]Laboratory of Malaria and Vector Research, National Institute of Allergy and Infectious Diseases, National Institutes of Health, Rockville, MD, USA

*Corresponding author:

niraj.tolia@nih.gov

9000 Rockville Pike, Building 29B, 4NN08

Bethesda, MD, 20892

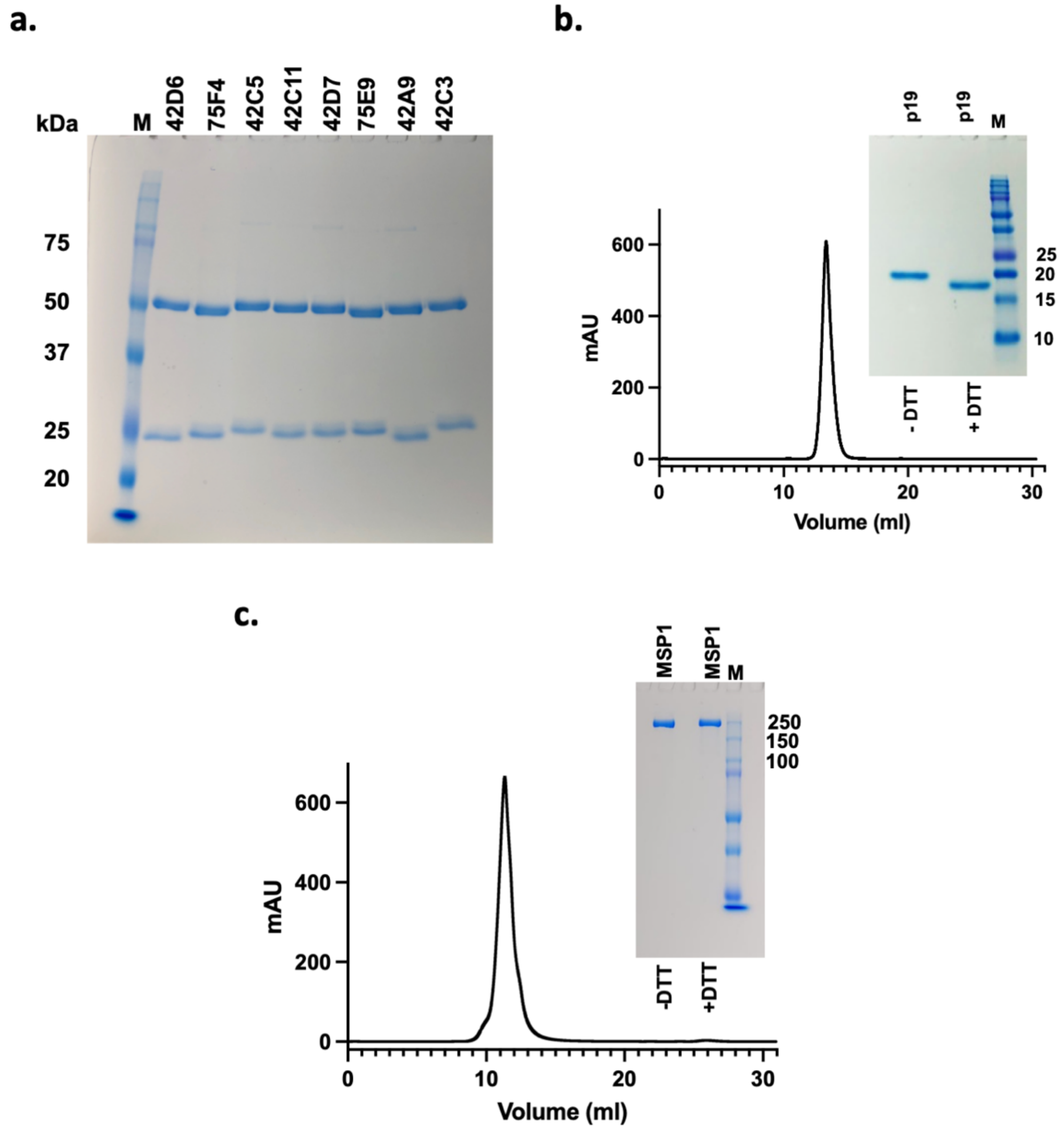

Supplementary Figure 1: Expression and purification of recombinant antigens and hmAbs. **a.** Reducing SDS-PAGE profile of expressed and purified hmAbs. **b.** The size exclusion chromatography (SEC) profile of recombinantly expressed p19 on Superdex 75 Increase 10/300 GL at 0.80 ml/min. **c.** SEC profile of recombinantly expressed full-length MSP-1 on Superdex 200 Increase 10/300 GL at 0.75 ml/min. The inset in **b.** and **c.** is an SDS-PAGE gel stained with Coomassie Blue showing the purity of the monomeric p19 (14.339 kDa) and full-length MSP-1

(193 kDa), respectively. SDS-PAGE gels stained with Coomassie Blue were cropped to areas of interest at time of image acquisition. Images were not further manipulated. The experiments were repeated at least three times with similar results. Source data are provided as a Source Data file.

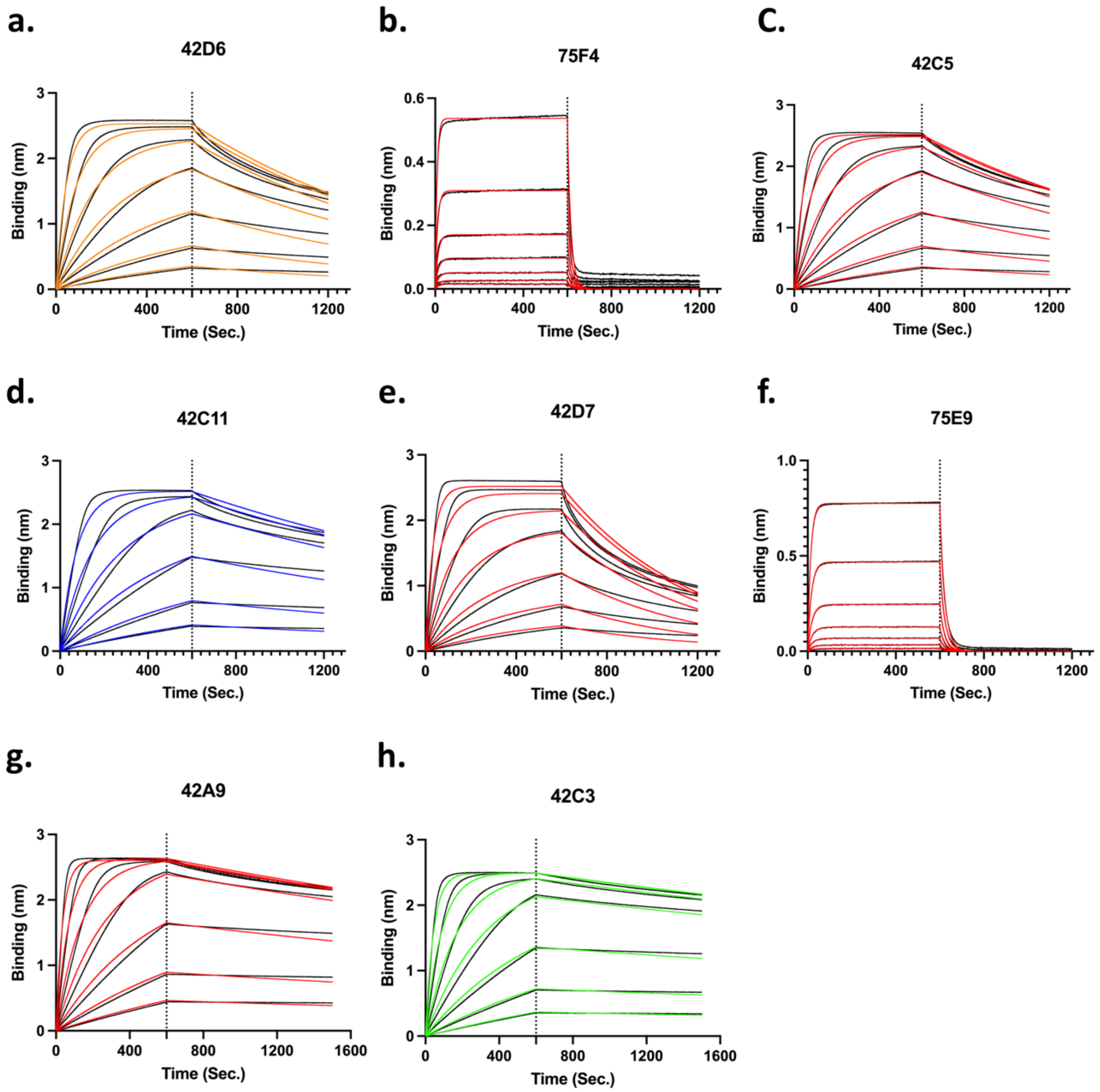

Supplementary Figure 2: Binding kinetics of anti-p19 mAbs. (a.-h.) Representative sensorgrams and 1:1 model best fits (orange, red, blue, and green) for Fab binding to p19, determined by biolayer interferometry. A 2-fold dilution series was used beginning at the following concentrations **a.** 100- 1.56 nM, **b.** 200- 3.13 nM, **c.** 100- 1.56 nM, **d.** 50- 1.56 nM, **e.** 100- 1.56 nM, **f.** 200-3.13 nM, **g.** 100- 1.56 nM, and **h.** 100- 1.56 nM. 1:1 model best fit for mAbs

42D6, 42C11, and 42C3 are colored in orange, blue, and green, respectively to be consistent with the color code throughout the manuscript. Source data are provided as a Source Data file.

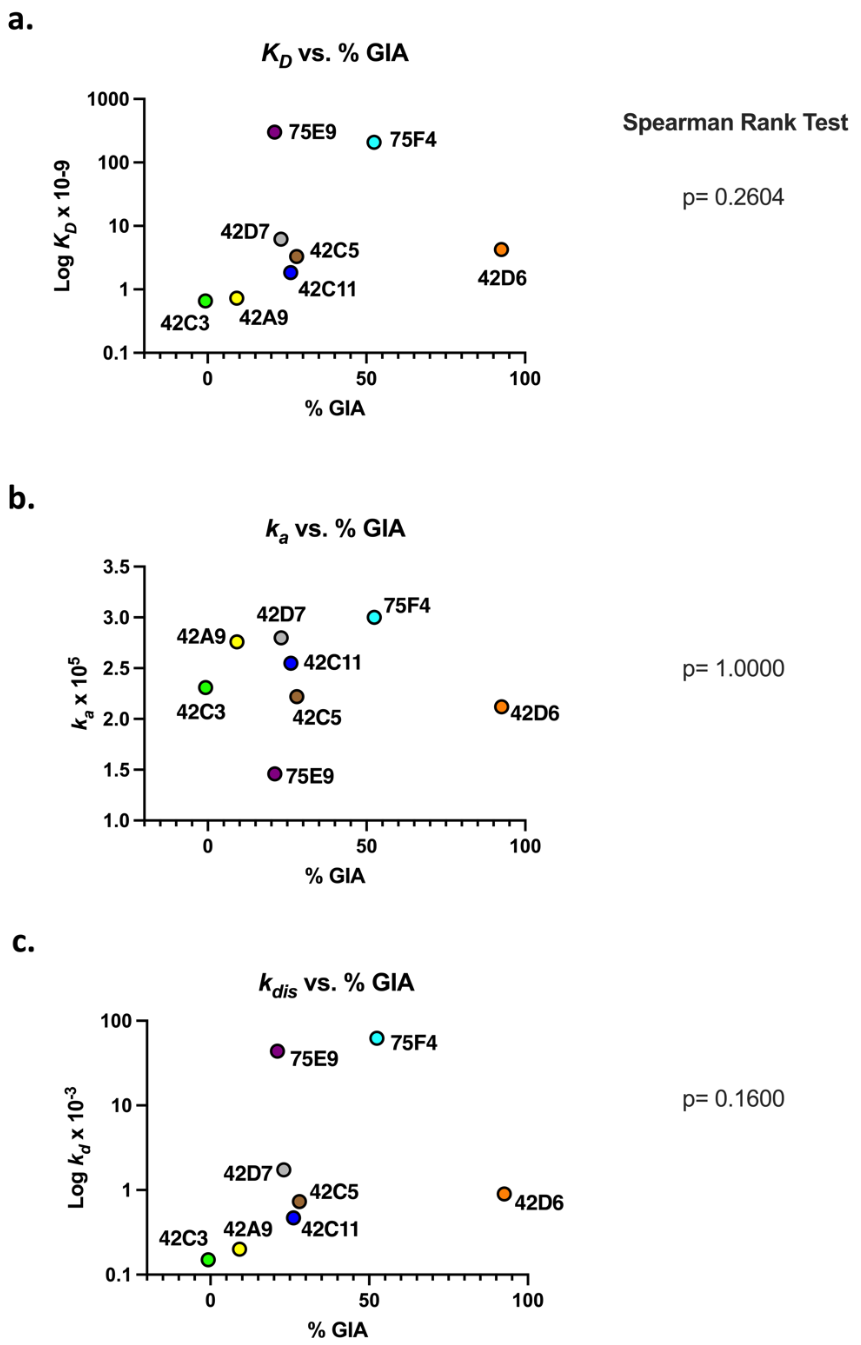

Supplementary Figure 3: Binding kinetics and GIA show no correlation. a. Dissociation constant (K_D), **b.** Association rate constant (k_a), and **c.** Dissociation rate constant (k_{dis}) plotted against % GIA for hmAbs. Kinetic data are as in Table 1 and % GIA value represents an average for each hmAb tested at 1.0 mg/ml against the *P. falciparum 3D7* blood stage in 5 independent assays. Source data are provided as a Source Data file.

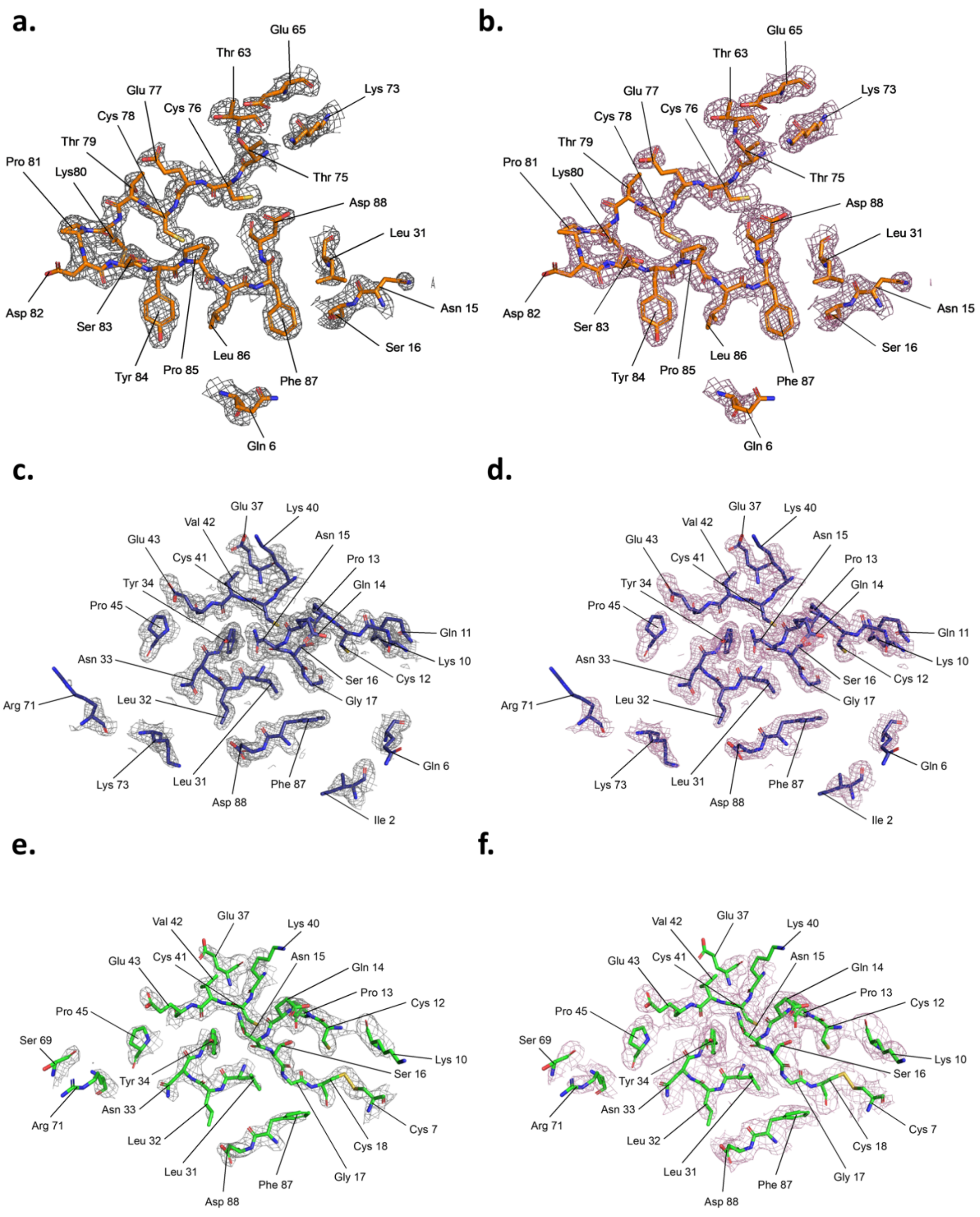

Supplementary Figure 4: Representative electron density of the 42D6, 42C11, and 42C3 epitopes on p19. Electron density for the **a.** 42D6, **c.** 42C11, and **e.** 42C3 epitope residues from a 2Fo-Fc map (gray mesh) contoured at 1.0 σ level (1.43 rmsd). Electron density for the **b.** 42D6, **d.** 42C11, and **f.** 42C3 epitope residues from a 2mFo-DFc composite omit map (pink mesh) contoured at 1.0 σ level (1.43 rmsd). Epitope residues are depicted as a stick model.

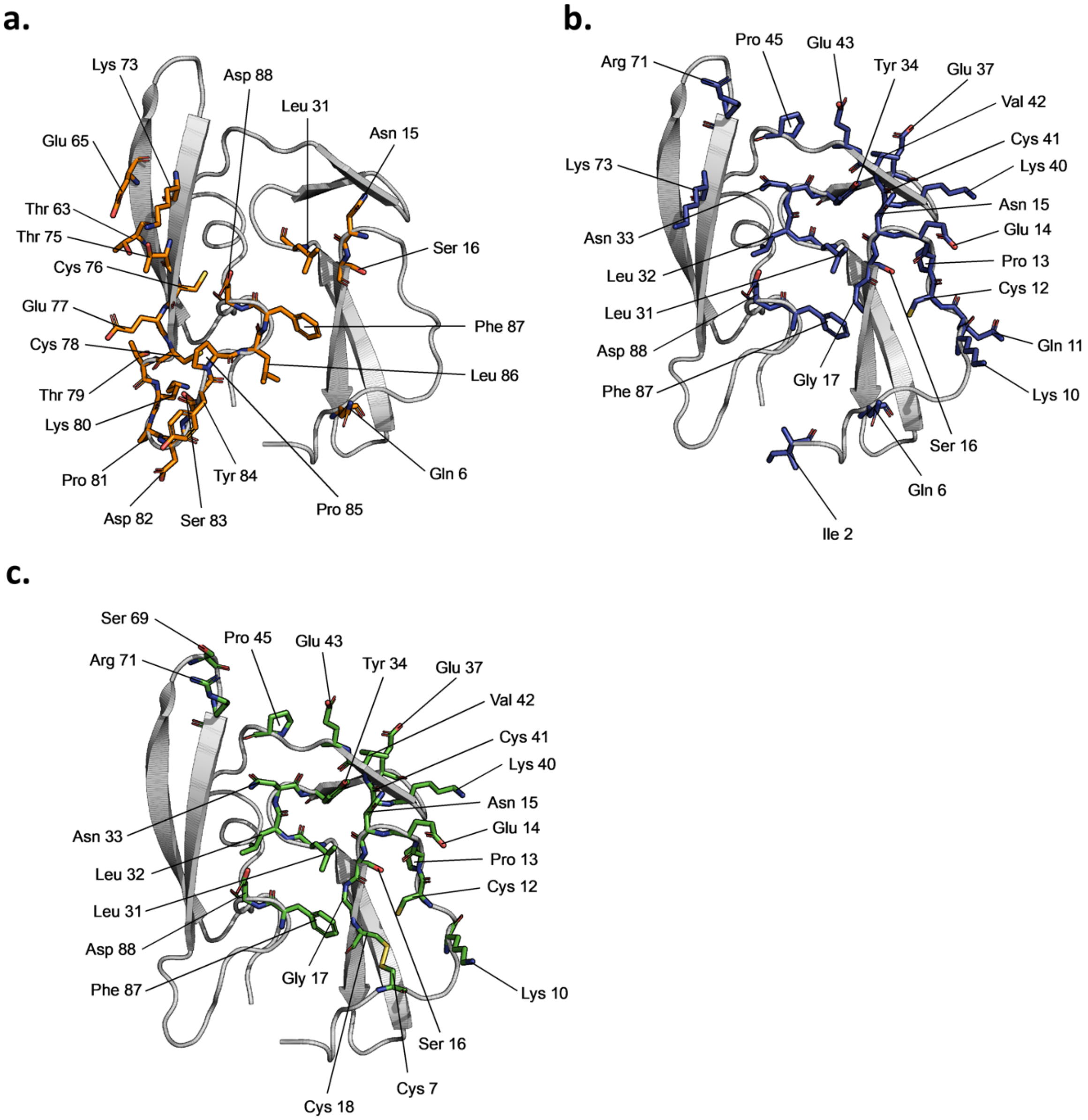

Supplementary Figure 5: Detailed view of the p19 epitopes for a. 42D6, b. 42C11 and c. 42C3 mAbs, respectively. Grey, p19; orange, p19 residues contacted by 42D6 heavy chain CDRs; blue, p19 residues contacted by 42C11 heavy and light chain CDRs; green, p19 residues contacted by 42C3 heavy and light chain CDRs.

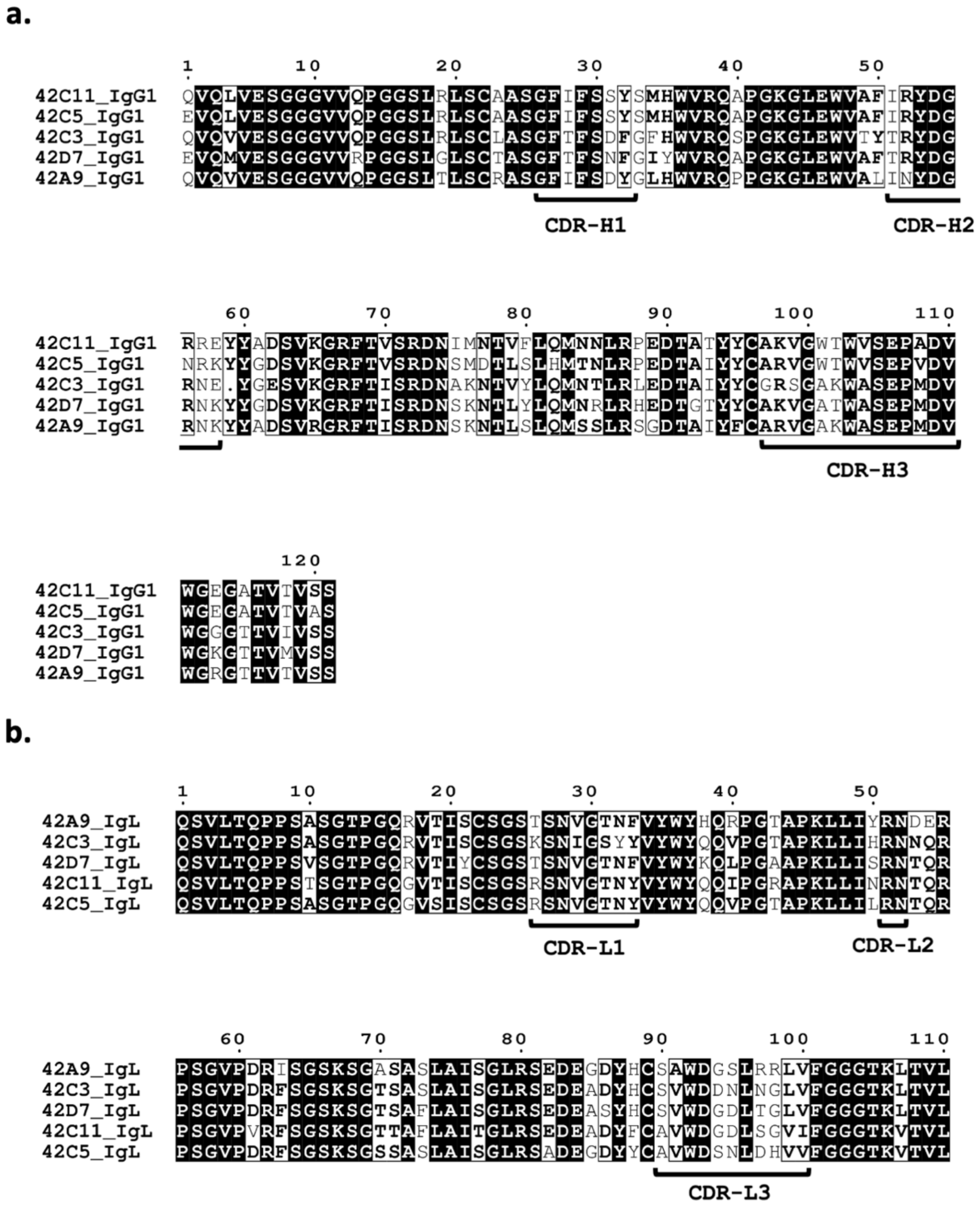

Supplementary Figure 6: hmAbs 42C11, 42C5, 42C3, 42D7, and 42A9 were isolated from the same individual and are likely clonally related. Amino acid sequence alignment of the variable region of **a.** heavy and **b.** light chains of hmAbs. CDRs are shown at the bottom of the alignment. Multiple sequence alignments were constructed using Clustal Omega/T-Coffee.

```
        1          11         21         31         41         51
Pf3D7   NISQHQCVKKQCPENSGCFRHLDEREECKCLLNYKQEGDKCVENPNPTCNENNGGCDADA
PfFUP   NISQHQCVKKQCPENSGCFRHLDEREECKCLLNYKQEGDKCVENPNPTCNENNGGCDADA
PfDd2   NISQHQCVKKQCPENSGCFRHLDEREECKCLLNYKQEGDKCVENPNPTCNENNGGCDADA
PfHB3   NISQHQCVKKQCPQNSGCFRHLDEREECKCLLNYKQEGDKCVENPNPTCNENNGGCDADA
PfFVO   NISQHQCVKKQCPQNSGCFRHLDEREECKCLLNYKQEGDKCVENPNPTCNENNGGCDADA
cons    *************:**********************************************

        61         71         81         91
Pf3D7   TCTEEDSGSSRKKITCECTKPDSYPLFDGIFCS
PfFUP   KCTEEDSGSNGKKITCECTKPDSYPLFDGIFCS
PfDd2   KCTEEDSGSNGKKITCECTKPDSYPLFDGIFCS
PfHB3   KCTEEDSGSNGKKITCECTKPDSYPLFDGIFCS
PfFVO   KCTEEDSGSNGKKITCECTKPDSYPLFDGIFCS
cons    .********. **********************
```

Supplementary Figure 7: Sequence alignment of p19 from various strains of *P. falciparum*. Polymorphic residues between strains are highlighted in red. Interface residues of p19 with 42D6 Fab are colored in blue. Multiple sequence alignments were constructed using Clustal Omega/T-Coffee.

**Pf**	**SHM**	**heavy chain**	**heavy chain**	**heavy chain**	**heavy chain**	**heavy chain**	**heavy chain**	**SHM**	**light chain**	**light chain**	**light chain**	**light chain**	**light chain**
**mAb (IgG)**	**(aa changes in heavy chain V region)**	**V-gene and allele**	**D-gene and allele**	**J-GENE and allele**	**CDR1**	**CDR2**	**CDR3**	**(aa changes in light chain V region)**	**V-GENE and allele**	**J-GENE and allele**	**CDR1**	**CDR2**	**CDR3**
75F4	17	IGHV4-4*07 F	IGHD3-16*02 F	IGHJ5*02 F	GGSIT NYY	IFGSGIT	ARSPPNPVFPRFDP	17	IGKV3-15*01 F	IGKJ4*01 F, or IGKJ4*02 (F)	QNIGTK	GA	QQYNDWPPVT
75E9	20	IGHV4-4*07 F	IGHD3-16*02 F	IGHJ5*02 F	GGSIS NYY	VFHTGIT	ARSPPNPVFPRFDP	19	IGKV3-15*01 F	IGKJ4*01 F, or IGKJ4*02 (F)	QNIGTK	GA	QQYSDWPPVT
42D7	16	IGHV3-30*02 F or IGHV3-30-5*02 F	IGHD1-26*01 F	IGHJ6*03 F	GFTFS NFG	TRYDGRNK	AKVGATWASEPMDV	18	IGLV1-47*01 F	IGLJ2*01 F, or IGLJ3*01 F or IGLJ3*02 F	TSNVG TNF	RN	SVWDGDLTGLV
42D6	12	IGHV4-4*07 F	IGHD3-22*01 F	IGHJ4*02 F	RGSIGS YY	VYSNGRA	AREIYYHDSTGSLYYFDY	16	IGKV3-15*01 F	IGKJ1*01 F	QTVSSS	GA	LQFNDWPPT
42C5	15	IGHV3-30*02 F or IGHV3-30-5*02 F	IGHD1-1*01 F	IGHJ6*04 F	GFIFSS YS	IRYDGNRK	ARVGWTWVSEPVDV	14	IGLV1-47*01 F	IGLJ2*01 F, or IGLJ3*01 F	RSNVG TNY	RN	AVWDSNLDHVV
42C3	18	IGHV3-30*02 F	IGHD3-16*01 F	IGHJ6*04 F	GFTFS DFG	TRYDGRN	GRSGAKWASEPMDV	9	IGLV1-47*01 F	IGLJ2*01 F or IGLJ3*01 F	KSNIGS YY	RN	SVWDDNLNGLV
42C11	13	IGHV3-30*02 F	IGHD3-9*01 F	IGHJ6*04 F	GFIFSS YS	IRYDGRRE	AKVGWTWVSEPADV	17	IGLV1-47*01 F	IGLJ2*01 F or IGLJ3*01 F	RSNVG TNY	RN	AVWDGDLSGVI
42A9	18	IGHV3-30*02 F or IGHV3-30-5*02 F	IGHD6-19*01 F	IGHJ6*04 F	GFIFSD YG	INYDGRNK	ARVGAKWASEPMDV	14	IGLV1-47*01 F	IGLJ2*01 F, or IGLJ3*01 F or IGLJ3*02 F	TSNVG TNF	RN	SAWDGSLRRLV
42A3	20	IGHV3-30*02 F or IGHV3-30-5*02 F	IGHD1-26*01 F	IGHJ6*04 F	GFTFS NYG	IQYDGRIT	GRVGATWASEPADV	19	IGLV1-47*01 F	IGLJ2*01 F, or IGLJ3*01 F or IGLJ3*02 F	RSNVG TNF	RN	SVLDGNLGGLV

Supplementary Table 1: V, D, and J gene information, CDR sequences, and the number of amino acid (aa) changes of cloned *Plasmodium falciparum* MSP1-specific mAbs.

**Contacts between p19 and 42D6**			
**Residues in p19**	**Interface with 42D6 chains**	**Residues in 42D6 Heavy chain**	**CDRs, Heavy chain**
Gln 6	Heavy	Gly 27	1
Asn 15	Heavy	Ser 28	1
Ser 16	Heavy	Gly 30	1
Leu 31	Heavy	Ser 31	1
Thr 63	Heavy	Tyr 32	1
Glu 65	Heavy	Tyr 33	1
Lys 73	Heavy	Tyr 52	2
Thr 75	Heavy	Ser 53	2
Cys 76	Heavy	Asn 54	2
Glu 77	Heavy	Arg 56	2
Cys 78	Heavy	Lys 73	
Thr 79	Heavy	Arg97	3
Lys 80	Heavy	Ile 99	3
Pro 81	Heavy	Tyr 100	3
Asp 82	Heavy	His 102	3
Ser 83	Heavy	Asp 103	3
Tyr 84	Heavy	Ser 104	3
Pro 85	Heavy	Thr 105	3
Leu 86	Heavy	Gly 106	3
Phe 87	Heavy	Ser 107	3
ASP 88	Heavy	Tyr 109	3

Supplementary Table 2: Contact residues between p19 and 42D6 mAb.

Contacts between p19 and 42C11					
**Residues in p19**	**Interface with 42C11 chains**	**Residues in 42C11 Heavy chain**	**CDRs, Heavy chain**	**Residues in 42C11 Light chain**	**CDRs, Light chain**
Ile 2	Heavy	Phe 50		Arg 26	1
Gln 6	Heavy	Arg 52	2	Ser 27	1
Lys 10	Heavy	Tyr 53	2	Gly 30	1
Gln 11	Heavy	Arg 56	2	Thr 31	1
Cys 12	Heavy	Arg 57	2	Asn 32	1
Pro 13	Heavy	Tyr 59		Tyr 33	1
Gln 14	Heavy, Light	Trp 101	3	Arg 51	2
Asn 15	Heavy, Light	Thr 102	3	Asn 52	2
Ser 16	Heavy	Trp 103	3	Gln 54	
Gly 17	Heavy	Val 104	3	Lys 67	
Leu 31	Heavy	Ser 105	3	Trp 92	3
Leu 32	Heavy, Light	Glu 106	3	Gly 94	3
Asn 33	Heavy, Light			Leu 96	3
Tyr 34	Heavy, Light			Ser 97	3
Glu 37	Light				
Lys 40	Light				
Cys 41	Light				
Val 42	Light				
Glu 43	Light				
Pro 45	Light				
Arg 71	Light				
Lys 73	Heavy				
Phe 87	Heavy				
Asp 88	Heavy				

Supplementary Table 3: Contact residues between p19 and 42C11 mAb.

Contacts between p19 and 42C3					
**Residues in p19**	**Interface with 42C3 chains**	**Residues in 42C3 Heavy chain**	**CDRs, Heavy chain**	**Residues in 42C3 Light chain**	**CDRs, Light chain**
Cys 7	Heavy	Tyr 50		Gly 30	1
Lys 10	Heavy	Arg 52	2	Ser 31	1
Cys 12	Heavy	Tyr 53	2	Tyr 32	1
Pro 13	Heavy	Asp 54	2	Tyr 33	1
Gln 14	Heavy, Light	Gly 55	2	Arg 51	2
Asn 15	Heavy, Light	Arg 56	2	Asn 52	2
Ser 16	Heavy	Glu 58		Asn 53	
Gly 17	Heavy	Lys 101	3	Gln 54	
Cys 18	Heavy	Trp 102	3	Lys 67	
Leu 31	Heavy	Ala 103	3	Trp 92	3
Leu 32	Light	Ser 104	3	Asp 94	3
Asn 33	Light	Glu 105	3	Asn 97	3
Tyr 34	Heavy, Light				
Glu 37	Light				
Lys 40	Light				
Cys 41	Light				
Val 42	Light				
Glu 43	Light				
Pro 45	Light				
Ser 69	Light				
Arg 71	Light				
Phe 87	Heavy				
Asp 88	Heavy				

Supplementary Table 4: Contact residues between p19 and 42C3 mAb.

	K_D (x 10^{-9} ± SEM M)	k_a (x 10^{5} ± SEM 1/Ms)	k_{dis} (x 10^{-3} ± SEM 1/s)	N
**T61K**	4.57 ± 0.05	2.16 ± 0.02	0.99 ± 0.01	3
**E65K**	25.68 ± 0.76	1.90 ± 0.01	4.87 ± 0.11	3
**L86F**	7.96 ± 0.15	2.75 ± 0.01	2.19 ± 0.04	3

Supplementary Table 5: Kinetic rate constants of binding for Fab fragment of 42D6 mAb to polymorphic variants of p19, as determined by BLI. Dissociation constant (K_D), Association rate constant (k_a), Dissociation rate constant (k_{dis}).
