# Supplementary material for: Neutralizing and interfering human antibodies define the structural and mechanistic basis for antigenic diversion
Source: Nat Commun. 2022 Oct 6;13:5888. doi: 10.1038/s41467-022-33336-3 (PMC9537153; doi:10.1038/s41467-022-33336-3)
Supplement: Supplementary file 3 — Reporting Summary [file 41467_2022_33336_MOESM3_ESM.pdf]

nature portfolio

Corresponding author(s): Niraj Tolia

Last updated by author(s): Aug 12, 2022

Reporting Summary

Nature Portfolio wishes to improve the reproducibility of the work that we publish. This form provides structure for consistency and transparency in reporting. For further information on Nature Portfolio policies, see our Editorial Policies and the Editorial Policy Checklist.

Statistics

For all statistical analyses, confirm that the following items are present in the figure legend, table legend, main text, or Methods section.

n/a	Confirmed	
☐	☒	The exact sample size (*n*) for each experimental group/condition, given as a discrete number and unit of measurement
☐	☒	A statement on whether measurements were taken from distinct samples or whether the same sample was measured repeatedly
☒	☐	The statistical test(s) used AND whether they are one- or two-sided<br>*Only common tests should be described solely by name; describe more complex techniques in the Methods section.*
☒	☐	A description of all covariates tested
☒	☐	A description of any assumptions or corrections, such as tests of normality and adjustment for multiple comparisons
☒	☐	A full description of the statistical parameters including central tendency (e.g. means) or other basic estimates (e.g. regression coefficient) AND variation (e.g. standard deviation) or associated estimates of uncertainty (e.g. confidence intervals)
☒	☐	For null hypothesis testing, the test statistic (e.g. *F*, *t*, *r*) with confidence intervals, effect sizes, degrees of freedom and *P* value noted<br>*Give P values as exact values whenever suitable.*
☒	☐	For Bayesian analysis, information on the choice of priors and Markov chain Monte Carlo settings
☒	☐	For hierarchical and complex designs, identification of the appropriate level for tests and full reporting of outcomes
☒	☐	Estimates of effect sizes (e.g. Cohen's *d*, Pearson's *r*), indicating how they were calculated

Our web collection on statistics for biologists contains articles on many of the points above.

Software and code

Policy information about availability of computer code

Data collection	UNICORN 7.3 software was used to collect FPLC data. FortéBio Data Acquisition 12.0 software was used to collect the bio-layer interferometry data. Gen5 3.08.01 software was used to collect the ELISA data.
Data analysis	- GraphPad Prism 9.2.0.<br>- FortéBio Data Analysis HT 12.0.<br>- XDS Package (version February 5, 2021), Phenix 1.16 and 1.20 (includes Phaser, AutoBuild, and Phenix.refine), Coot 0.9.8.1 and PyMol 2.5.1 curated by SBGrid.<br>- IMGT/V-QUEST online https://www.imgt.org/IMGTindex/V-QUEST.php.<br>- Clustal Omega online https://www.ebi.ac.uk/Tools/msa/clustalo/<br>- T-Coffee online https://www.ebi.ac.uk/Tools/msa/tcoffee/

For manuscripts utilizing custom algorithms or software that are central to the research but not yet described in published literature, software must be made available to editors and reviewers. We strongly encourage code deposition in a community repository (e.g. GitHub). See the Nature Portfolio guidelines for submitting code & software for further information.

Data

Policy information about availability of data

All manuscripts must include a data availability statement. This statement should provide the following information, where applicable:

- Accession codes, unique identifiers, or web links for publicly available datasets
- A description of any restrictions on data availability
- For clinical datasets or third party data, please ensure that the statement adheres to our policy

All data generated or analysed during this study are included in this published article, source data file and supplementary information files. Atomic coordinates and structure factors have been deposited in the Protein Data Bank with PDB IDs 8DFG, 8DFH and 8DFI. Source data are provided with this paper.

Human research participants

Policy information about studies involving human research participants and Sex and Gender in Research.

Reporting on sex and gender	Details of the study cohort, sample processing, and hmAb isolation have been described previously.30, 43
Population characteristics	Details of the study cohort, sample processing, and hmAb isolation have been described previously.30, 43
Recruitment	Details of the study cohort, sample processing, and hmAb isolation have been described previously.30, 43
Ethics oversight	The hmAbs characterized in this study were isolated form PBMCs obtained from subjects enrolled in an observational cohort study conducted in the rural community of Kalifabougou, Mali. Details of the study cohort, sample processing, and hmAb isolation have been described previously.30, 43 The Ethics Committee of the Faculty of Medicine, Pharmacy, and Dentistry at the University of Sciences, Technique, and Technology of Bamako, and the Institutional Review Board of the National Institute of Allergy and Infectious Diseases, National Institutes of Health, approved this study. Written informed consent was obtained from adult participants and from the parents or guardians of participating children. The cohort study is registered in the ClinicalTrials.gov database (NCT01322581).

Note that full information on the approval of the study protocol must also be provided in the manuscript.

Field-specific reporting

Please select the one below that is the best fit for your research. If you are not sure, read the appropriate sections before making your selection.

☒ Life sciences ☐ Behavioural & social sciences ☐ Ecological, evolutionary & environmental sciences

For a reference copy of the document with all sections, see nature.com/documents/nr-reporting-summary-flat.pdf

Life sciences study design

All studies must disclose on these points even when the disclosure is negative.

Sample size	Sample size calculation is not applicable to the experimental design of the growth inhibition assay, structural studies or biophysical approaches included in this manuscript.
Data exclusions	No data exclusion.
Replication	The number of biological and technical replicates for each assay are shown in related figure legends and source data file. All attempts at replication were successful.
Randomization	No randomization. Randomization is not applicable to the experimental design of the growth inhibition assay, structural studies or biophysical approaches included in this manuscript.
Blinding	No blinding. Blinding is not applicable experimental design of the growth inhibition assay, structural studies or biophysical approaches included in this manuscript.

Reporting for specific materials, systems and methods

We require information from authors about some types of materials, experimental systems and methods used in many studies. Here, indicate whether each material, system or method listed is relevant to your study. If you are not sure if a list item applies to your research, read the appropriate section before selecting a response.

Materials & experimental systems

n/a	Involved in the study
☐	☒ Antibodies
☐	☒ Eukaryotic cell lines
☒	☐ Palaeontology and archaeology
☒	☐ Animals and other organisms
☒	☐ Clinical data
☒	☐ Dual use research of concern

Methods

n/a	Involved in the study
☒	☐ ChIP-seq
☒	☐ Flow cytometry
☒	☐ MRI-based neuroimaging

Antibodies

Antibodies used

The goat anti-human Ab conjugated to HRP (catalog no.: 109-035-098, Polyclonal, Lot: 146365) purchased from Jackson ImmunoResearch (West Grove, PA, USA) was used as a secondary antibody in ELISA. mAbs 42D6, 75F4, 42C5, 42C11, 42D7, 75E9, 42A9, 42C3, and TB31F were expressed in HEK293 cells (Expi293F™ cells).

Validation

mAbs 42D6, 75F4, 42C5, 42C11, 42D7, 75E9, 42A9, and 42C3 were validated in this study by sequencing, ELISA, structural studies, binding kinetics, and functional activity. The goat anti-human Ab conjugated to HRP (catalog no.: 109-035-098, Polyclonal, Lot: 146365) purchased from Jackson ImmunoResearch (West Grove, PA, USA) was validated in numerous previous scientific publications and by the manufacturer.

Eukaryotic cell lines

Policy information about cell lines and Sex and Gender in Research

Cell line source(s)

Expi293F™ cells were purchased from Thermo Fisher Scientific.

Authentication

Expi293F™ cells were authenticated for Viability and Mycoplasma (Mycoplasma qPCR Assay) by Thermo Fisher Scientific. Expi293F™ cells were more than 90 % viable and negative for Mycoplasma.

Mycoplasma contamination

The cells were not tested for Mycoplasma contamination. No previous case of contamination was ever detected in our laboratory.

Commonly misidentified lines
(See ICLAC register)

No commonly misidentified lines were used.
